# Pharmaceutical Treatments for Typical CKD Comorbidities

**DOI:** 10.3390/biomedicines14020401

**Published:** 2026-02-10

**Authors:** Manuela Di Lauro, Annalisa Noce

**Affiliations:** 1 Department of Systems Medicine, University of Rome Tor Vergata, 00133 Rome, Italy; dilauromanuela@gmail.com; 2 UOSD Nephrology and Dialysis, Policlinico Tor Vergata, 00133 Rome, Italy

Chronic kidney disease (CKD) represents a major global health challenge due to its high prevalence in the general population and its substantial impact in terms of morbidity, mortality, and healthcare costs [1]. CKD should not be considered an isolated condition but rather a complex systemic syndrome characterized by multiple metabolic disturbances and a wide range of comorbidities. Globally these factors significantly contribute both to disease progression toward end-stage renal disease (ESRD) and to an increased risk of cardiovascular (CV) events and all-cause mortality [2].

Among the most frequently associated comorbidities are diabetes mellitus and arterial hypertension, representing the leading causes of renal impairment [3]. Diabetes mellitus promotes the progression of nephropathy through mechanisms involving chronic hyperglycaemia, oxidative stress, and inflammation, whereas arterial hypertension accelerates glomerular and vascular damage, establishing a vicious cycle that further worsens renal function.

Another critical aspect of CKD is protein–energy wasting (PEW), a multifactorial condition resulting from reduced protein and caloric intake, chronic inflammation, oxidative stress, metabolic acidosis, and increased protein catabolism [4]. PEW contributes to the development of uremic sarcopenia, characterized by progressive loss of muscle mass and strength, leading to impaired physical performance and an increased risk of falls, hospitalization, and mortality [5].

Disorders of mineral and bone metabolism (CKD–MBD) represent another hallmark complication of CKD. These abnormalities include disturbances in calcium, phosphate, vitamin D, and parathyroid hormone homeostasis, leading to bone fragility, vascular calcification, and an increased CV risk [6]. Metabolic acidosis, resulting from the reduced renal capacity to excrete acid load, frequently coexists with CKD–MBD and contributes to muscle protein breakdown, bone demineralization, and accelerated CKD progression [7].

Endothelial dysfunction represents a key pathophysiological mechanism underlying the high CV burden observed in CKD patients. The endothelium plays a central role in maintaining vascular homeostasis by regulating vascular tone, platelet aggregation, leukocyte adhesion, inflammation, and thrombosis. In CKD, progressive renal impairment is associated with profound structural and functional alterations of the vascular endothelium, which contribute not only to accelerated atherosclerosis and CV disease but also to the progression of renal dysfunction itself [8].

CKD patients frequently exhibit an immuno-fragile profile characterized by the coexistence of immune dysfunction, chronic inflammation, accelerated aging [9], and consequently, a short-life expectancy. Uremia-associated immune dysregulation leads to defects in both innate and adaptive immunity, resulting in increased susceptibility to infections, reduced vaccine responsiveness, and a higher risk of malignancy. At the same time, persistent low-grade inflammation, called micro-inflammation, contributes to immune exhaustion and accelerates CV and renal disease progression [10]. This state of immune fragility is further exacerbated by metabolic disturbances, oxidative stress, and the accumulation of uremic toxins [11].

Within this complex and multifaceted clinical scenario, the identification and implementation of novel pharmacological therapies are of paramount importance for improving the clinical management of CKD patients. Nevertheless, the complexity of CKD requires an approach that extends beyond pharmacological treatment alone. Non-pharmacological interventions (including personalized nutritional strategies, tailored physical exercise programs, lifestyle modification, and psychological support) are essential components of a truly effective and comprehensive management strategy [12]. A holistic and multidisciplinary approach allows simultaneous targeting of the multiple dimensions of CKD, ultimately improving not only patient survival but also health-related quality of life.

The aim of this Special Issue is, therefore, to provide an up-to-date overview of the most innovative pharmacological and non-pharmacological therapies for CKD management, with particular emphasis on integrated and personalized approaches.

Specifically, Albakr et al. (contribution 1) examined in a retrospective study the efficacy and safety of finerenone in patients with non-diabetic CKD, a setting for which data are still limited. The Authors highlighted a significant reduction in albuminuria, accompanied by a significant improvement in systolic and diastolic blood pressure, suggesting a possible therapeutic role in this population as well.

Moreover, Ceprián et al. (contribution 2) studied immunological status and frailty in CKD patients undergoing various renal replacement treatments (haemodialysis—HD, peritoneal dialysis, renal transplantation), analyzing their impact on mortality. Immunophenotypic analysis revealed a reduction in lymphocyte populations and an increase in pro-inflammatory monocytes, particularly in HD patients, who also had the highest prevalence of frailty. The Authors highlight the importance of developing personalized therapeutic interventions in this patient population.

Furthermore, three interesting reviews have explored other aspects typical of CKD. In particular, Lazarou et al. (contribution 3) highlighted the importance of performing renal biopsies as a fundamental tool in the management of kidney transplant recipients, as they allow for accurate assessment of graft health and identification of subclinical pathological alterations undetectable with common clinical and laboratory tests. Early identification of these conditions allows for timely therapeutic interventions, particularly optimization of immunosuppressive therapy, with a positive impact on graft function and survival.

Danneel et al. (contribution 4) analyze how drugs, such as ACE inhibitors, angiotensin II receptor blockers, and sodium–glucose cotransporter type 2 inhibitors, play a key role in reducing intraglomerular pressure and renal inflammation. Furthermore, the Authors point out the importance of a multidisciplinary approach, emphasizing how greater collaboration between nephrologists, pharmacologists, and dietitians can improve patients’ self-management of the disease.

Finally, the review by Marrone et al. (contribution 5) analyses the main diagnostic methods used to evaluate the endothelial dysfunction, distinguishing between direct and indirect techniques, recognizing the direct study of coronary endothelial function as the diagnostic gold standard. Furthermore, the main therapeutic strategies to address endothelial dysfunction in CKD patients are discussed, including both pharmacological treatments, such as sodium–glucose cotransporter 2 inhibitors and mineralocorticoid receptor antagonists, and innovative non-pharmacological interventions (such as the consumption of extra virgin olive oil, adherence to a plant-dominant low-protein diet, an adaptive physical activity program, and, finally, ketoanalogue administration).
